# Hypersensitivity Reaction to Tirzepatide With Demonstrated Tolerance to Semaglutide: A Case Report

**DOI:** 10.1002/ccr3.73183

**Published:** 2026-07-22

**Authors:** Cameron Nejat, Robert Lin, Daniel Addo, Kristyl Cuenca‐Sisko, Mary Lee‐Wong

**Affiliations:** ^1^ Department of Public and Ecosystem Health Cornell University Ithaca New York USA; ^2^ New Jersey Medical School, Rutgers Biomedical and Health Sciences Newark New Jersey USA; ^3^ Division of Allergy and Immunology Weill Cornell Medical College New York New York City USA; ^4^ New York Allergy and Sinus Centers New York New York City USA; ^5^ Division of Allergy and Immunology Icahn School of Medicine at Mount Sinai New York New York City USA

**Keywords:** glucagon‐like peptide‐1, glucose‐dependent insulinotropic polypeptide, obesity medication hypersensitivity, semaglutide, tirzepatide

## Abstract

Hypersensitivity reactions to glucagon‐like peptide‐1 receptor agonists (GLP‐1RAs) are uncommon but clinically significant given their expanding use for obesity and type 2 diabetes. Tirzepatide, a dual GLP‐1/glucose‐dependent insulinotropic polypeptide (GIP) receptor agonist, and reports of allergic reactions remain limited. We present this case to highlight the diagnostic utility of intradermal testing and supervised drug challenge in confirming drug‐specific hypersensitivity and identifying a safe therapeutic alternative within the same drug class. We report a 25‐year‐old woman with class I obesity (BMI 32.25) who developed hypersensitivity reactions after dose escalation of tirzepatide initiated for weight management. She tolerated 3 months of sequential dose escalations from 2.5 mg through 7.5 mg without adverse reaction, losing approximately 20 lbs. during this period. Following her first 10 mg dose, she developed episodic lip angioedema and generalized urticaria over the subsequent 4 days, without respiratory or gastrointestinal symptoms. Symptoms persisted despite intramuscular diphenhydramine, a 5‐day course of prednisone, and two emergency department visits with intravenous steroids, ultimately resolving only after tirzepatide discontinuation. Laboratory evaluation conducted approximately 8 months prior to the reaction, following a separate self‐limited episode of lip swelling unrelated to tirzepatide, had demonstrated normal total IgE, normal C1 esterase inhibitor protein and function, and normal complement levels, effectively excluding hereditary and acquired angioedema. *H. pylori* urea breath testing obtained, and allergy evaluation (which excluded food and seasonal allergies) was negative. Percutaneous skin testing to tirzepatide was negative; however, intradermal testing at 1:10 dilution was positive (8 mm wheal, 8 mm flare), confirming drug hypersensitivity. Intradermal testing to semaglutide was negative. A supervised subcutaneous challenge with semaglutide 0.25 mg was well tolerated, and the patient was cleared to initiate semaglutide under endocrinology oversight. The patient has since continued semaglutide without recurrence. This case illustrates tirzepatide‐specific hypersensitivity and highlights the diagnostic value of intradermal testing and supervised challenge in guiding safe continuation of therapy with alternative GLP‐1RAs.

## Key Clinical Message

Tirzepatide can cause drug‐specific hypersensitivity confirmed by intradermal testing, even when percutaneous skin testing is negative. In patients requiring GLP‐1 receptor agonist therapy, structured skin testing and supervised drug challenge can identify a safe alternative agent, enabling continuation of medically necessary treatment.

## 1 Introduction

Glucagon‐like peptide‐1 receptor agonists (GLP‐1RAs) are now cornerstone therapies for obesity and type 2 diabetes, given their effects on weight and glycemia [1]. Tirzepatide is the first once‐weekly dual incretin agonist targeting both the GIP and GLP‐1 receptors, and clinical trials have shown dose‐dependent reductions in HbA1c and body weight with a safety profile broadly similar to GLP‐1RAs [2]. As use expands from diabetes into obesity and cardiovascular/metabolic indications, clinicians will encounter a wider spectrum of adverse events [3, 4]. These adverse events may include an increased incidence of hypersensitivity reactions and allergies to GLP‐1 receptor agonists.

Gastrointestinal symptoms remain the most frequent reactions to tirzepatide; by contrast, hypersensitivity reactions are uncommon and not well characterized [5]. Prior literature mentions immediate anaphylaxis and local injection site reactions; however, there is a paucity of information regarding the mechanism of these reactions [6, 7]. There are no standardized, validated skin‐testing protocols published for GLP‐1 receptor agonists [8]. Cross‐reactivity within this class remains uncertain. Reported prick/intradermal testing concentrations are drawn from individual case reports [8, 9]. Evaluation is patient‐focused and often confirmed with a supervised graded challenge [9]. These gaps complicate decisions about discontinuation versus switching within the incretin class.

We present a case of tirzepatide hypersensitivity confirmed by intradermal testing. Subsequently, this patient was negative for both intradermal semaglutide testing and graded challenge. This practical approach provides an alternative incretin therapy for patients with tirzepatide hypersensitivity.

## 2 Case History/Examination

A 25‐year‐old woman with class I obesity (height 161.3 cm, weight 83.9 kg; BMI 32.25 kg/m^2^) and no prior drug allergies initiated once‐weekly tirzepatide for weight management. She tolerated the medication over 3 months without reported adverse reactions during treatment, losing approximately 9.1 kg.

After tolerating 3 months of subsequent dose escalations (from 2.5 mg ➔ 5 mg ➔ 7.5 mg ➔ 10 mg), she administered her first 10 mg dose. For the subsequent 4 days after the first 10 mg injection, the patient developed chronic lip angioedema and generalized urticaria. She did not experience dyspnea, wheezing, syncope, or gastrointestinal symptoms. She was treated at urgent care with intramuscular diphenhydramine and a five‐day course of prednisone 20 mg. However, lip swelling and tingling persisted despite corticosteroid therapy. Over two emergency department visits, she received intravenous steroids, diphenhydramine, was observed overnight, and was discharged with an epinephrine auto‐injector and a methylprednisolone dose pack. Tirzepatide was discontinued, and no further angioedema occurred thereafter. She subsequently required daily antihistamine therapy for symptom management. When the patient abruptly discontinued cetirizine, 48 h prior to allergy test, this resulted in severe generalized pruritus and burning sensations. This suggests her antihistamine regimen may have been suppressing a more systemic reactive process.

## 3 Differential Diagnosis, Investigations and Treatment

Approximately 8 months before the tirzepatide‐linked reaction, and following a self‐limited episode of lip swelling, the patient underwent evaluation for possible hereditary or autoimmune angioedema. Laboratory evaluation demonstrated a normal total IgE (62 IU/mL), normal C1 esterase inhibitor protein (37 mg/dL) with functional activity greater than 110%, normal complement C1q (15.6 mg/dL), and normal complement C4 (29 mg/dL), effectively excluding hereditary and acquired angioedema. Complement C3 was mildly elevated at 183 mg/dL (reference 82–167 mg/dL), which may reflect a low‐level systemic inflammatory state. Serum protein electrophoresis showed no M‐spike and all fractions were within normal limits. These results can be seen in Table 1 below.

**TABLE 1:** Summary of laboratory and allergy testing.

Hereditary/Autoimmune Angioedema Workup—approximately 8 months prior to reaction		
Test	Result	Normal range
Total IgE	62 IU/mL	6–495 IU/mL
C1 esterase inhibitor, serum	37 mg/dL	21–39 mg/dL
C1 esterase inhibitor, functional activity	> 110%	> 67% (Normal)
Complement C1q	15.6 mg/dL	10.3–20.5 mg/dL
Complement C4	29 mg/dL	12–38 mg/dL
Complement C3	183 mg/dL ↑	82–167 mg/dL
Serum protein electrophoresis—M‐spike	Not observed	Not Observed
Serum protein electrophoresis—All fractions	Within normal limits	Within normal limits

Initial allergy evaluation		
Test	Result	Normal range
Vital signs	Stable	—
Percutaneous skin testing (environmental and food allergens)	Negative	—
*H. pylori* urea breath test	Not detected	Not detected

Follow up allergy testing visit—drug testing		
Test	Result	Interpretation
Vital signs	Stable	—
**Percutaneous skin testing (SPT)**		
Positive histamine control (SPT)	8 mm wheal/15 mm flare	Valid
Negative saline control (SPT)	0 mm wheal/2 mm flare	Valid
Percutaneous skin test—Tirzepatide	0 mm wheal/2 mm flare	Negative
Percutaneous skin test—Semaglutide	0 mm wheal/2 mm flare	Negative
**Intradermal testing (ID)**		
Positive histamine control (ID)	15 mm wheal/40 mm flare	Valid
Negative saline control (ID)	0 mm wheal/2 mm flare	Valid
Intradermal test—Tirzepatide (1:10 dilution)	8 mm wheal/8 mm flare	Positive—hypersensitivity
Intradermal test—Semaglutide (1:10 dilution)	0 mm wheal/2 mm flare	Negative
Subcutaneous challenge—Semaglutide 0.25 mg	No reaction	Tolerated—monitored ×1 h

Following the initial workup, in which hereditary and acquired angioedema were excluded and total IgE was within normal limits, the first episode was attributed to idiopathic angioedema. The patient had no prior history of drug allergy, atopic disease, or recurrent allergic episodes beyond this isolated event. When lip angioedema and urticaria recurred in clear temporal association with tirzepatide dose escalation, a full repeat systemic workup was deferred given the established baseline labs and identifiable drug trigger; evaluation was focused on confirmatory drug skin testing to characterize the hypersensitivity and identify a safe alternative.

At the initial allergy evaluation, vital signs were stable and antihistamines had been withheld prior to testing. Previous percutaneous skin testing on environmental and food allergens was negative. An *H. pylori* urea breath test was obtained per chronic spontaneous urticaria guidelines, as the infection is a recognized systemic urticaria trigger independent of gastrointestinal symptoms; the result was negative. These results can be seen in Table 1 below.

**FIGURE 1:**
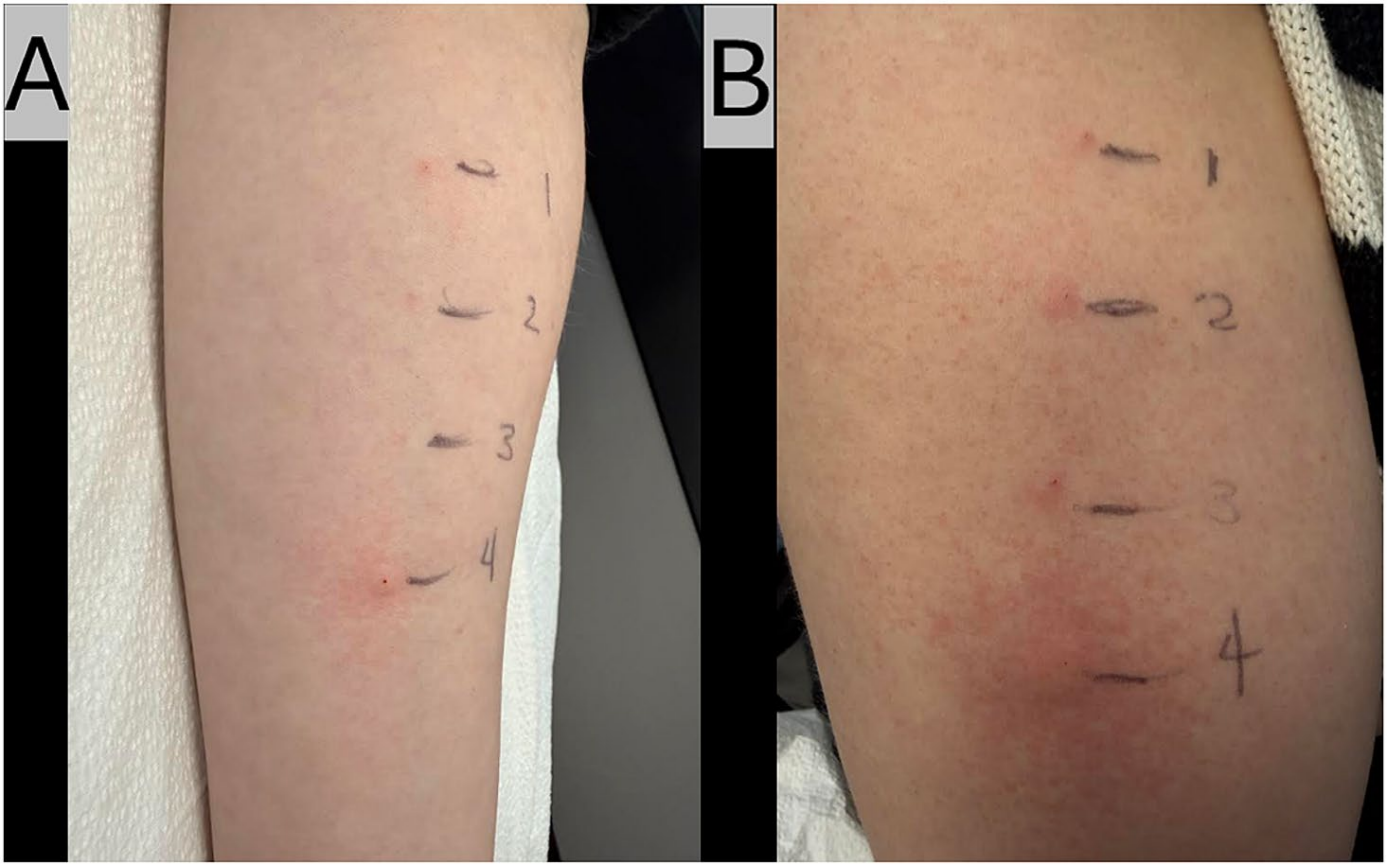
(A, B) Allergy testing results to GLP‐1 receptor agonists. (A) Percutaneous skin testing (SPT) and (B) intradermal testing (ID). Sites 1–4: Semaglutide, tirzepatide, negative saline control, and positive histamine control, respectively.

At the subsequent allergy testing visit, vital signs remained stable and antihistamines had been withheld prior to testing. Percutaneous skin testing to tirzepatide was negative, with a valid positive histamine control. Intradermal testing to tirzepatide at 1:10 dilution was positive (8 mm wheal, 8 mm flare), consistent with a hypersensitivity pattern. Intradermal testing to semaglutide at 1:10 dilution was negative. These results can be seen in Table 1 below. SPT and IT testing can be seen in Figure 1, All testing was read approximately 20 min after administration. Subsequently, a subcutaneous challenge with semaglutide was administered; the patient was monitored for 1 h without incident and discharged in stable condition.

## 4 Outcomes and Follow Up

She was advised to initiate semaglutide under endocrinology oversight and to avoid tirzepatide due to the positive reaction to intradermal Tirzepatide. The patient has since continued semaglutide without recurrence.

## 5 Discussion

Glucagon‐like peptide‐1 (GLP‐1) is a naturally occurring incretin hormone released from the gut after food intake.1 It plays an important role in glucose regulation by stimulating insulin secretion, suppressing glucagon release, slowing gastric emptying, and reducing appetite [10]. However, native GLP‐1 has a very short half‐life of only a few minutes because it is rapidly broken down by the enzyme dipeptidyl peptidase‐4 (DPP‐4) and quickly cleared by the kidneys, limiting its direct therapeutic use [11].

To overcome these limitations, several GLP‐1 receptor agonists (GLP‐1 RAs) have been developed, such as exenatide, liraglutide, and semaglutide [10, 11]. These drugs are structurally modified to resist DPP‐4 degradation and to remain in the circulation for longer periods [10, 11]. Most traditional GLP‐1 RAs are selective for the GLP‐1 receptor and largely replicate the physiological effects of endogenous GLP‐1, including appetite suppression and improved glycemic control [11].

**FIGURE 2:**
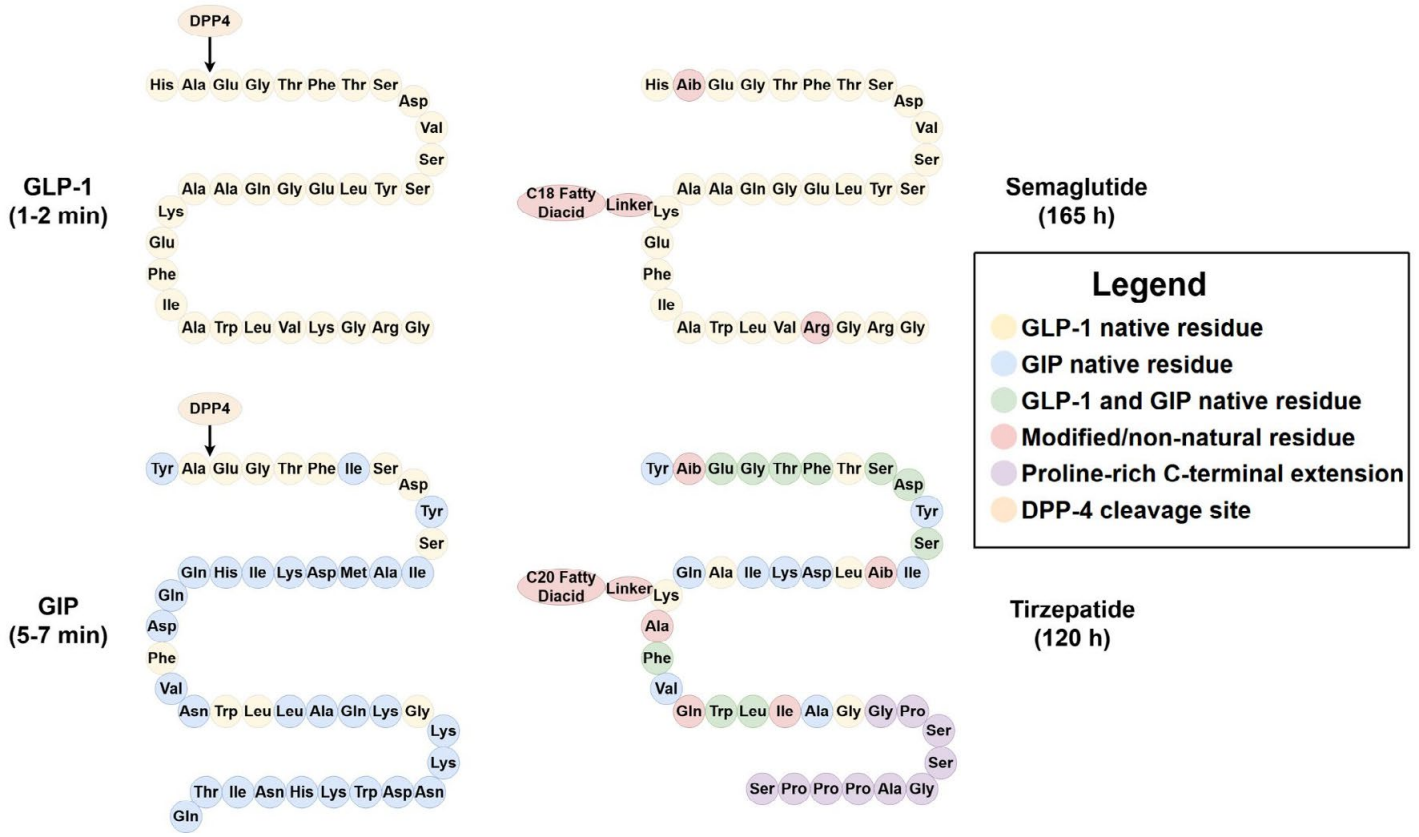
Amino acid sequence of GLP‐1, GIP, semaglutide, and tirzepatide and their relative half‐lives [11, 12, 13, 14, 15, 16].

Semaglutide and tirzepatide have been structurally engineered to have a much longer duration of action, as seen in Figure 2 [2, 11, 12, 13, 14, 15, 16]. DPP‐4 resistance in semaglutide and tirzepatide is achieved through substitution of alanine with 2‐aminoisobutyric acid (Aib) at position 8 [17, 18]. Also, a C18 and C20 fatty diacid conjugated to Lys20, in semaglutide and tirzepatide respectively, enables reversible albumin binding and prolonged systemic exposure [17, 18]. This albumin binding slows drug clearance and reduces renal filtration, resulting in a half‐life of approximately 5 and 7 days, for tirzepatide and semaglutide, respectively, and enabling once‐weekly dosing [19, 20].

Tirzepatide differs from conventional GLP‐1 receptor agonists in both mechanism of action and pharmacokinetic design [2]. Unlike other GLP‐1, based therapies, tirzepatide is a dual agonist that activates both the glucose‐dependent insulinotropic polypeptide (GIP) receptor and the GLP‐1 receptor [2]. GIP is another incretin hormone that enhances insulin secretion and may improve insulin sensitivity and lipid metabolism [2]. By targeting both receptors simultaneously, tirzepatide produces broader metabolic effects than GLP‐1‐only agents, contributing to greater reductions in blood glucose levels and body weight observed in clinical studies [2].

Since the patient tolerated semaglutide without symptoms, this pattern raises the possibility that her reaction to tirzepatide was driven by a tirzepatide‐specific factor, potentially related to its GIP‐receptor agonist component or other unique structural features, rather than a generalized GLP‐1 mediated hypersensitivity. However, this remains speculative, and alternative explanations, like excipients or non–receptor‐specific immunogenicity, cannot be excluded.

Hypersensitivity reactions to tirzepatide were reported in 3.2% of tirzepatide‐treated patients in placebo‐controlled trials, with higher rates among patients who developed anti‐tirzepatide antibodies, suggesting a broadly immune‐mediated mechanism [16].

The positive intradermal test to tirzepatide, in the setting of urticaria and angioedema, is most consistent with IgE‐mediated drug hypersensitivity. While total serum IgE was within normal limits at baseline, measured approximately 8 months prior to tirzepatide initiation, this does not exclude subsequent drug‐specific IgE sensitization developing over the course of treatment, as intradermal testing is a more sensitive method for detecting localized IgE‐mediated responses. Although urticaria and angioedema are classically associated with Type I hypersensitivity, the four‐day symptom course following dose escalation after months of prior tolerance is atypical for an immediate reaction and raises the possibility of a delayed or mixed mechanism, as delayed‐onset cutaneous reactions following prolonged drug exposure have been described in the literature. Such delayed reactions may reflect a T cell‐mediated component, in which repeated drug exposure drives sensitization and subsequent effector responses upon dose escalation. Formal mechanistic testing was not performed and the precise mechanism remains uncharacterized in this case.

This case highlights three clinical lessons: tirzepatide hypersensitivity can emerge after months of tolerated dose escalation; percutaneous testing alone may be insufficient, making intradermal testing essential when suspicion remains; and tirzepatide hypersensitivity does not preclude use of alternative GLP‐1RAs. Structured skin testing and supervised challenge enabled safe continuation of therapy with semaglutide, offering a practical management pathway as incretin use expands.

## Author Contributions

**Cameron Nejat:** writing – original draft, writing – review and editing, visualization, methodology, conceptualization. **Mary Lee‐Wong:** methodology, writing – review and editing, supervision, conceptualization. **Robert Lin:** conceptualization, investigation, supervision, methodology, writing – review and editing. **Daniel Addo:** writing – original draft, writing – review and editing. **Kristyl Cuenca‐Sisko:** investigation, conceptualization.

## Funding

The authors have nothing to report.

## Consent

Written informed consent was obtained from the patient for publication of this case report and any accompanying images, with all personally identifying information removed in accordance with ICMJE guidelines; a copy of the written consent is available for review by the Editor‐in‐Chief upon request.

## Conflicts of Interest

The authors declare no conflicts of interest.

## Data Availability

Data sharing is not applicable to this article as no datasets were generated or analyzed during the current study.
